# Real-World Experience with Long-Acting Injectable Cabotegravir/Rilpivirine in HIV Patients with Unsuppressed Viral Load

**DOI:** 10.3390/v17091254

**Published:** 2025-09-17

**Authors:** Marcello Trizzino, Luca Pipitò, Pierluigi Francesco Salvo, Federica Zimmerhofer, Andrea Cicero, Gianmaria Baldin, Claudia Conti, Claudia Gioè, Simona Di Giambenedetto, Antonio Cascio

**Affiliations:** 1 Infectious and Tropical Diseases Unit and Sicilian Regional Reference Center for the Fight Against AIDS, AOU Policlinico “P. Giaccone,” 90133 Palermo, Italy; marcello.trizzino@policlinico.pa.it (M.T.); lucapipito@gmail.com (L.P.); federica.zimmerhofer@community.unipa.it (F.Z.); andrea.cicero@community.unipa.it (A.C.); claudia.conti@community.unipa.it (C.C.); claudia.gioe@policlinico.pa.it (C.G.); 2 Palermo Fast-Track City, Casa dei Diritti, 90100 Palermo, Italy; 3 Department of Health Promotion, Mother and Child Care, Internal Medicine, and Medical Specialties “G D’Alessandro,” University of Palermo, 90133 Palermo, Italy; 4 Division of Infectious Diseases, Department of Medical and Surgical Sciences, Agostino Gemelli University Hospital Foundation IRCCS, 00168 Rome, Italy; pierluigifsalvo@gmail.com (P.F.S.); gianmaria.baldin@policlinicogemelli.it (G.B.); simona.digiambenedetto@policlinicogemelli.it (S.D.G.); 5 Department of Safety and Bioethics, Infectious Diseases Section, Catholic University of the Sacred Heart, 00168 Rome, Italy

**Keywords:** HIV, long-acting, off label, cabotegravir, rilpivirine

## Abstract

Long-acting injectable cabotegravir/rilpivirine (CAB/RPV-LA) is currently approved as a maintenance therapy for people with HIV (PWH) who are virologically suppressed. However, growing real-world evidence highlights its potential role in more complex viremic populations traditionally considered ineligible. We present a case series of eight PWH treated at two tertiary centers in Italy, all of whom faced persistent viremia, adherence difficulties, malabsorption syndromes, or psychosocial barriers. Following the switch to CAB/RPV-LA, all patients, despite heterogeneous clinical profiles and baseline virological status, achieved and maintained virologic suppression, demonstrated improved adherence, and experienced no serious adverse events.

## 1. Introduction

The global fight against Human Immunodeficiency Virus (HIV) has seen remarkable progress over the past four decades, transforming a once fatal diagnosis into a manageable chronic condition with the advent of effective antiretroviral therapy (ART) [1]. Despite significant advancements, including highly potent and well-tolerated oral regimens, challenges persist in achieving universal viral suppression and preventing HIV-related morbidity and mortality. New strategies are continually being developed to address ongoing barriers to effective treatment, particularly those related to adherence and the complex needs of patients. In many settings, barriers to sustained adherence to ART are multifactorial, involving stigma, mental illness, substance use, structural and socioeconomic inequities, and treatment fatigue [2]. Long-acting (LA) injectables like cabotegravir/rilpivirine (CAB/RPV) offer a promising solution by reducing dosing frequency from daily pills to bi-monthly or monthly intramuscular injections, thereby directly addressing adherence challenges and mitigating social barriers associated with daily pill-taking [3,4]. CAB is an integrase strand transfer inhibitor. It works by binding to the HIV integrase enzyme and blocking the strand transfer step of viral DNA integration into the host genome, which is an essential process for viral replication. RPV is a non-nucleoside reverse transcriptase inhibitor (NNRTI). It binds directly to reverse transcriptase, causing a conformational change that inhibits the enzyme’s activity. This prevents the transcription of viral RNA into DNA, thereby halting replication [3,4].

However, CAB/RPV-LA was initially approved by regulatory bodies, including the Food and Drug Administration and European Medicines Agency, as a complete regimen for virologically suppressed people with HIV (PWH), primarily as a switch strategy from an existing oral ART, provided there are no known or suspected CAB or RPV resistance mutations [5,6]. These criteria largely excluded high-need populations who could potentially benefit most from its LA nature. These include individuals who are viraemic [7,8], those with malabsorption syndromes [9], patients with documented poor adherence to oral ART [4,9], and those who are unable to take oral medications due to various reasons [7,9]. Emerging real-world evidence and expert consensus increasingly support the expanded use of CAB/RPV-LA in these “difficult-to-treat” populations [4,7]. Here, we reported a case series of PWH switched to CAB/RPV-LA under challenging settings.

## 2. Materials and Methods

This work is based on a real-world case series designed to illustrate the use of CAB/RPV-LA in challenging settings. Eight representative cases were retrospectively selected from patients followed between 2021 and 2025 at two tertiary-level Infectious Diseases units in Italy: the Infectious and Tropical Diseases Unit of the “Paolo Giaccone” University Hospital in Palermo (cases from 1 to 6), and the Infectious Diseases Unit of the “Agostino Gemelli” University Hospital IRCCS in Rome (cases 7 and 8). Selection criteria included: history of virologic failure or persistent viremia; severe adherence challenges; relevant comorbidities affecting drug absorption, cognitive function, or social support; and transition to CAB/RPV-LA. Each patient received 600 mg of CAB and 900 mg of RPV per dose.

Data were retrieved from electronic health records, including demographics, HIV history, prior ART, viral load and CD4+ counts, resistance testing, adherence assessments, and clinical outcomes following LA therapy initiation. All data were anonymized in accordance with applicable privacy regulations.

## 3. Cases

### 3.1. Case 1

A 54-year-old Italian male was diagnosed with HIV in 2019, presenting as a late presenter with wasting syndrome. At diagnosis, his HIV-RNA was 729,236 copies/mL, and his CD4 count was 79 cells/mm^3^. Genotypic resistance testing (GRT) revealed a wild-type virus (subtype B), fully susceptible to all ART classes. His medical history was notable for two episodes of syphilis and an mpox virus infection. Initial ART consisted of oral darunavir/cobicistat/tenofovir alafenamide/emtricitabine (DRV/c/TAF/FTC). Despite initiating ART, the patient struggled to achieve consistent viral suppression. His complex medical history included a diagnosis of Inflammatory Bowel Disease (IBD) in February 2022. In December 2023, he experienced a severe IBD flare, characterized by 8–10 bloody bowel movements per day and a C-reactive protein level exceeding 200 mg/L, necessitating the initiation of infliximab. This severe gastrointestinal pathology significantly compromised the absorption of his oral ART, leading to recurrent viremia despite presumed good adherence to the oral regimen. Given the persistent virologic non-suppression attributed to malabsorption secondary to severe IBD, a decision was made to switch the patient to CAB/RPV-LA. The rationale was to bypass the impaired gastrointestinal absorption by providing direct intramuscular drug delivery. Remarkably, after initiating CAB/RPV-LA, the patient rapidly achieved and maintained virologic suppression, with HIV RNA consistently below 50 copies/ml. However, therapeutic drug monitoring (TDM) of CAB and RPV plasma levels consistently showed values below the established therapeutic cut-offs (CAB cut-off: 1120 ng/mL; RPV cut-off: 32 ng/mL), but dosing regimen was not changed. This presented a “pharmacokinetic paradox”: profound virological suppression despite apparent “subtherapeutic” drug levels. No adverse effects were reported during the follow-up period apart from injection-site reactions.

### 3.2. Case 2

A 43-year-old Italian man with HIV infection diagnosed in February 2016 had a history of uncontrolled HIV viral load. At the time of diagnosis, his CD4 count was 269 cells/mm^3^ and his viral load was >2,000,000 copies/mL. No opportunistic infections were detected. Baseline GRT, performed using the ViroSeq HIV-1 Genotyping System, revealed a subtype B virus with the T215Y mutation (on NRTI) and the Y188C mutation (on non-nucleoside reverse transcriptase inhibitor, NNRTI). The patient has consistently been characterized by poor adherence to both outpatient follow-ups and ART. In February 2022, he reported a therapeutic interruption lasting approximately one and a half years. His therapeutic history began in 2016 with a protease inhibitor (PI) combined with an old integrase inhibitor (INI) regimen. Subsequently, following a prolonged interruption, he resumed therapy with DRV/c/TAF/FTC. Furthermore, his medical history was characterized by two subsequent hospitalizations for Pneumocystis pneumonia and anxiety-depressive syndrome with episodes of major depression, requiring numerous psychiatric consultations. These symptoms were interpreted as a reaction to the experience of his HIV diagnosis. The patient also reported progressive socio-relational withdrawal, with significant difficulties sharing daily life moments with friends and family members. Such psychosocial challenges, including the impact of stigma, are well-documented barriers to optimal ART adherence. Despite poor ART adherence, a GRT performed in February 2023 (with HIV-RNA at 8560 copies/mL) showed a subtype B virus sensitive to all current drug classes. However, the patient again reported poor and discontinuous adherence to his oral therapy. By January 2025, he presented with a residual viremia of 2300 copies/mL. A subsequent NGS-based GRT on HIV-RNA performed under drug pressure confirmed the absence of significant resistance mutations. Considering his persistent poor adherence to oral therapy and the significant psychosocial burdens he faced, an extensive counselling process was undertaken, and he ultimately agreed to start CAB/RPV-LA. Currently, the patient has received three administrations and has demonstrated full adherence to the new regimen, respecting the scheduled administration windows. His plasma viral load is now suppressed (<20 copies/mL). He continues his psychiatric treatment and reports a subjective improvement in his quality of life, which he attributes directly to the reduced psychological burden associated with daily ART management. No adverse effects were reported during the follow-up period apart from injection-site reactions.

### 3.3. Case 3

A 63-year-old woman, originally from the Ivory Coast, was diagnosed with HIV infection in 2012. She was initially followed at another centre, and no baseline clinical data or GRT results are available. She experienced several antiretroviral regimens based on PI, INI, and NNRTI. All regimen changes were motivated by reported gastrointestinal intolerance (stomach ache, abdominal bloating), leading to irregular adherence and frequent virological failures. She was successfully treated for Helicobacter pylori gastritis, and several gastroscopies and biopsies did not reveal abnormalities. Over time, several other therapeutic regimens were attempted, including DRV/c/TAF/FTC, doravirine/tenofovir disoproxil/lamivudine (DOR/TDF/3TC), dolutegravir/abacavir/lamivudine (DTG/ABC/3TC), and bictegravir/tenofovir alafenamide/emtricitabine (BIC/TAF/FTC). The patient exhibited progressive psychological resistance to therapy, refused further specialist (gastroenterology) consultations, and rejected any diagnostic or therapeutic proposals outside of infectious diseases. Execution of a GRT under virological failure (HIV Viral load 18,900 copies/mL) showed a CRF06_cpx virus sensitivity to all drug classes. Due to the impossibility of ensuring continuity with oral therapy, CAB/RPV-LA was initiated intramuscularly. From the first administration, the patient did not report any gastrointestinal symptoms. Her viremia was suppressed (<20 copies/mL), and adherence to the bi-monthly administrations remained optimal over the following year. A viral blip of 142 copies/mL was observed after 10 months, followed by resuppression without any change in therapy. No adverse effects were reported during the follow-up period apart from injection-site reactions.

### 3.4. Case 4

A 37-year-old Italian male was diagnosed with HIV in 2019 following a probable acute retroviral syndrome in 2018. Initially, he was followed at a different center, but he was later transferred due to work relocation. The patient, a university-educated public health official, exhibited strong socio-cultural engagement and excellent adherence to care. At diagnosis, HIV-RNA was 308,322 copies/mL, and CD4 count was 293 cells/mm^3^. His medical history includes arterial hypertension, bronchial asthma, grade I obesity, gastritis, and a family history of diabetes. Despite strict adherence, virological suppression was never fully achieved, and HIV viral load ranged from 72 copies/mL to 1850 copies/mL with multiple oral regimens between 2019 and 2024, including BIC/FTC/TAF, DRV/c/TAF/FTC, DRV/c + DOR + DTG, DOR + DTG + fostemsavir, and DTG + TDF/FTC. Resistance testing on proviral DNA in February 2023 revealed only secondary protease mutations (L10V, K20R, M36I, L63T, L89M), with no major resistance-associated mutations in reverse transcriptase or integrase, and a viral subtype F1. The genotype was interpreted as wild type. Persistent low-level viremia despite optimized therapy suggested a pharmacokinetic issue, likely related to impaired absorption secondary to gastrointestinal comorbidities. Consequently, in October 2024, the patient was transitioned to CAB/RPV-LA. At 9-month follow-up, sustained virological suppression (HIV-RNA < 50 copies/mL) was observed. No adverse effects were reported during the follow-up period apart from injection-site reactions.

### 3.5. Case 5

A 38-year-old Italian male was diagnosed with HIV infection in October 2023 following reactivation of herpes zoster and subsequent constitutional symptoms, including a 7 kg weight loss over three months. Baseline HIV-RNA was 995,000 copies/mL and CD4 count of 115 cells/mm^3^. GRT showed a wild-type virus (subtype B) with no resistance mutations. BIC/TAF/FTC was initiated immediately. The initial treatment response was robust, characterized by a rapid decrease in viral load and progressive CD4 recovery. However, from early 2024 onward, the patient experienced persistent low-level viremia, with HIV-RNA levels consistently ranging between 61 and 84 copies/mL, never achieving complete suppression (<50 copies/mL) despite excellent adherence and regular follow-up. Recognizing the consistent low-level viremia despite an effective oral regimen and good observed adherence, a decision was made to switch the patient to CAB/RPV-LA. CAB/RPV-LA therapy led to undetectable viral suppression (<20 copies/mL). No adverse effects were reported during the follow-up period apart from injection-site reactions.

### 3.6. Case 6

A 49-year-old Italian male was diagnosed with HIV in 2002, with a baseline CD4 count of 300 cells/mm^3^. Over more than two decades, he experienced frequent ART switches due to significant intolerance and adverse effects, despite achieving early virological and immunological control with a PI-based regimen. Subsequent regimens, including DTG/ABC/3TC, BIC/TAF/FTC, DTG/RPV, and DRV/c/TAF/FTC, were discontinued due to various side effects such as dyspepsia, insomnia, and dizziness. He remained on unboosted atazanavir with TAF/FTC for at least four years. His medical history was notable for an HCV genotype 1b infection that was successfully treated with sofosbuvir-velpatasvir. Despite access to effective ART, the patient displayed fluctuating viremia suggestive of adherence challenges, particularly evident in 2023–2024 with viral loads varying from <20 to 272,000 copies/mL. GRT in October 2024 showed no significant resistance mutations (subtype B). Coinciding with viral rebound, the patient developed new-onset psychiatric symptoms, including insomnia, nightmares, somnambulism, obsessive-compulsive behaviors, and severe anxiety, attributed in part to high viremia and ART changes. Psychiatric evaluations led to a diagnosis of chronobiological disruption and anxiety with obsessive features. Given recurrent ART intolerance, poor adherence driven by psychological and somatic symptoms, and the unsustainable nature of oral therapy, the patient was transitioned in December 2024 to CAB/RPV-LA. By May 2025, the patient had achieved full viral suppression, with HIV RNA becoming undetectable (<20 copies/mL). No adverse effects were reported during the follow-up period.

### 3.7. Case 7

A 59-year-old Italian male, born in October 1964, in Italy, with a self-reported history of men who have sex with men behaviour, received his HIV diagnosis on 29 December 2020. At the time of diagnosis, he presented as a late-stage patient, characterized by a significant viral burden with an HIV-RNA viral load of 325,500 copies/mL and a severely compromised immune status, indicated by a CD4 count of 25/mm^3^. This clinical picture was further complicated by the presence of a pulmonary Mycobacterium avium complex infection. Due to the acute presentation and immediate therapeutic focus, GRTs were not available. His medical history was otherwise notable for arterial hypertension, which constituted a relevant comorbidity requiring ongoing management. The patient’s ART commenced in January 2021 with a regimen of TDF/FTC/DTG. However, this regimen was regrettably discontinued in June 2021, a mere five months later, primarily due to identified drug–drug interactions. Subsequently, from June 2021 until October 2022, he was transitioned to DTG + DRV + ritonavir (r). Throughout this period, despite various efforts, the patient consistently exhibited only moderate adherence to his prescribed oral ART. This suboptimal adherence was largely attributable to significant socioeconomic factors, which presented persistent barriers to regular medication intake and consistent engagement with care. Considering his reported “pill fatigue”, a common challenge among long-term ART recipients, and the compounding difficulties posed by his disadvantaged socioeconomic context, a strategic decision was made to transition the patient to CAB/RPV-LA. The initial doses of CAB/RPV-LA were meticulously administered on 27 October 2022, followed by subsequent scheduled injections on 24 November 2022, 19 January 2023, and 12 April 2023, establishing a consistent regimen. At the point of CAB/RPV-LA initiation (20 October 2022), the patient’s viral load was measured at 86 copies/mL. His CD4 count stood at 166/mm^3^, with a corresponding CD8 count of 331/mm^3^. A rapid and encouraging virological response was observed shortly after the commencement of the injectable regimen. His HIV-RNA quickly declined to 40 copies/mL by 9 November 2022, and achieved complete undetectability (<30 copies/mL) by 24 November 2022. This impressive viral suppression was consistently maintained across all subsequent follow-up assessments, including those on 9 December 2022, 16 March 2023, and 11 April 2023, as well as over an extended period through 12 July 2024, and 2 December 2024. Concurrently, his immunological status showed a robust recovery, with his CD4 count progressively increasing from 166/mm^3^ at baseline to a peak of 405/mm^3^ by 3 December 2024, reflecting significant immune reconstitution. The CD8 count also demonstrated a positive trend, rising from 331/mm^3^ to 651/mm^3^ over the same timeframe. Throughout the entire follow-up period on CAB/RPV-LA, no adverse events were reported apart from injection-site reactions.

### 3.8. Case 8

A 52-year-old Italian woman, diagnosed with HIV in May 1997 (baseline HIV-RNA: 16,800 copies/mL; CD4 count: 328/mm^3^), had a history of Pneumocystis pneumonia, intravenous drug use, depression, and residual motor deficits following a prior ischemic stroke. These factors contributed to longstanding challenges with ART adherence. Her ART history was fragmented, with multiple interruptions and regimen changes over more than two decades. Initial ART, including lamivudine, zidovudine, and indinavir, was administered from June 1997 to July 1998. She re-engaged with care in October 2015 and received various oral regimens, including TDF/FTC/DRV/r, TAF/FTC + RAL, and TDF/FTC + RAL, with inconsistent virological suppression, likely due to poor adherence and social instability. In October 2022, given her suboptimal virological control and difficulty adhering to daily oral ART, she was transitioned to CAB/RPV-LA as a salvage approach. No oral lead-in was used. Initial injections were administered in October, November 2022, followed by bimonthly dosing. Before the initiation of CAB/RPV-LA in October 2022, her HIV-RNA was 46 copies/mL. Her CD4 and CD8 counts were 476/mm^3^ and 1074/mm^3^, respectively. Virological monitoring revealed transient viral blips (up to 1271 copies/mL in March 2023), but the patient ultimately achieved and maintained durable virological suppression (<30 copies/mL) from April 2023 through the most recent follow-up in June 2025. Immunologically, the patient experienced a significant initial dip in CD4 and CD8 counts following the first injection (CD4: 241/mm^3^ and CD8: 686/mm^3^ in November 2022), which subsequently recovered. Her CD4 count, initially 476/mm^3^ before CAB/RPV-LA, fluctuated but showed a remarkable upward trend, reaching 823/mm^3^ by July 2024, and settling at 755/mm^3^ by June 2025. Similarly, her CD8 count, starting at 1074/mm^3^, also demonstrated sustained improvement, peaking at 2117/mm^3^ by July 2024 and at 1682/mm^3^ by June 2025. No adverse events were reported during the follow-up period apart from injection-site reactions.

## 4. Discussion

CAB/RPV-LA has revolutionized HIV treatment by allowing PWH to discontinue daily oral regimens in favor of periodic intramuscular injections [10]. However, to date, the CAB/RPV-LA approach is approved exclusively for individuals with sustained viral suppression, and there is a lack of sufficient evidence regarding its use in other clinical settings where it may reveal its full therapeutic potential. Furthermore, multi-level strategies to support CAB/RPV implementation for PWH without virological suppression are required, which may necessitate additional resources in some settings to implement safely and effectively, and to eliminate outer-context barriers, including prior authorizations and specialty pharmacy restrictions [11]. In the Italian ARCA (Antiviral Response Cohort Analysis) Cohort of PWH, 44.5% (229) were eligible for CAB/RPV-LA, and compared with ineligible individuals, they received a lower number of previous regimens and were on current NNRTIs [12]. Iannone et al. reported data about 21 PWH who started CAB/RPV-LA, despite ineligibility for unsuppressed viral loads (median 66 cps/mL, IQR 40–215), and 10 (47.6%) achieved viral suppression. They calculated an overall probability of discontinuation of 14.9% at week 52 [13]. A retrospective observational study at the University of California–San Diego Owen Clinic showed promising results for the use of CAB/RPV-LA in 62 PWH with adherence difficulties to oral therapy. More than 85% of PWH with a history of adherence challenges maintained viral suppression at 48 weeks. Thirteen participants were prescribed additional ART [14]. Also, Gandhi et al. described the use of CAB/RPV-LA in 57 PWH with uncontrolled viremia. Of these, 54 had viral suppression at a median of 33 days [15]. The efficacy of CAB/RPV-LA was also reported in a limited cohort of pediatric population with unsuppressed viral load, including those with perinatally acquired infection, who have long faced challenges with medication adherence due to multiple factors (poorly palatable formulations, adverse side effects, and psychological barriers) [16].

We report our experience with eight PWH whose clinical features differ from those of the typical candidates eligible for CAB/RPV-LA (Table 1). They had complex clinical and treatment histories and initiated CAB/RPV-LA despite the absence of sustained viral suppression at baseline. Remarkably, all cases achieved and maintained virologic suppression, a feat not previously observed. These cases provide an opportunity to explore the application of CAB/RPV-LA across diverse clinical scenarios. CAB/RPV-LA represents an important therapeutic option in patients for whom daily oral therapy is not feasible, such as those with swallowing difficulties, malabsorption, or poor adherence to oral regimens. The injectable formulation allows sustained drug exposure with bi-monthly dosing, reducing pill burden and supporting adherence. In the first case, CAB/RPV-LA led to virologic suppression in a patient with IBD who, despite optimal adherence to oral therapy, had never achieved complete viral suppression. Given the persistent virologic non-suppression attributed to malabsorption secondary to severe IBD, a decision was made to switch the patient to CAB/RPV-LA. The rationale was to bypass the impaired gastrointestinal absorption by providing direct intramuscular drug delivery. The rapid and sustained virological suppression observed in this patient, despite persistently low TDM levels of CAB and RPV, challenges conventional pharmacokinetic thresholds for long-acting ART. TDM guidelines for CAB/RPV-LA acknowledge that wide inter-individual variability exists and that TDM should be interpreted cautiously, particularly in challenging scenarios [17]. Cossu et al. observed a lower interindividual variability in CAB plasma trough concentrations than rilpivirine in a cohort of 76 PWH [18]. However, TDM may help identify patients at risk of virological failure; however, reliable thresholds are lacking [19]. Sunagawa et al. reported a case of a late-presenter patient with miliary and meningeal tuberculosis, histoplasmosis, and bowel dysmotility, who started a conventional ART regimen via nasogastric tube, which resulted in intolerability, with severe emesis following each administration. This patient was switched to CAB/RPV-LA and intravenous AZT, and because of drug–drug interaction with rifampin, TDM was performed, showing optimal concentration of CAB and RPV below the limit of quantitation [20]. Psychiatric and psychological disorders related to HIV and stigma, which may compromise adherence, were observed in case 2 and represent another scenario in which CAB/RPV-LA may be advantageous. CAB/RPV-LA can be a life-changing intervention for individuals with a complex history of poor adherence driven by significant psychosocial and psychiatric comorbidities. Despite multiple oral regimen failures and the development of opportunistic infections due to inconsistent adherence, this patient achieved durable viral suppression and reported a marked improvement in quality of life. Kilcrease et al. described three cases of LA therapy use in patients with poor adherence. They described a young woman with perinatally acquired HIV with cognitive delay/limitations and very poor adherence to ART related to cognitive/developmental delay, lack of social support, housing instability, transportation difficulties, poverty, intermittent phone service, lack of childcare, pill burden, medication side effects, and internalized stigma, treated with CAB/RPV and ibalizumab-LA, and two cases of young women, one with cognitive limitations and one with nonadherence, that started CAB/RPV-LA that led to an undetectable VL (<20 copies/mL). All reached sustained virological suppression [21]. Poor tolerability of oral regimens, due to psychological factors, social determinants, adverse effects, or pill fatigue, was evident in cases 3, 6, 7, and 8, further supporting the utility of CAB/RPV-LA in such contexts. These cases highlight that traditional oral ART approaches were unsustainable in some scenarios due to a combination of real and perceived intolerances and the overwhelming impact of psychiatric symptoms on daily pill-taking. Persistent low-level viremia, as documented in cases 4, 5, and 7, may constitute another indication for transitioning to CAB/RPV-LA. In all reported cases, initiation of CAB/RPV-LA, without the need for an oral lead-in phase, and consistent adherence to the injection schedule resulted in stable viral suppression. Notably, patients who previously struggled with adherence to oral regimens demonstrated excellent compliance with injectable therapy. Potential explanations for low-level viremia include suboptimal adherence, subclinical malabsorption, drug–drug interactions, and pharmacokinetic variability. These challenges may be overcome by CAB/RPV-LA.

In addition, new LA regimens are being investigated in PWH who have unsuppressed viral loads. For instance, the combination of CAB and lenacapavir demonstrated a 94% virologic suppression rate in a cohort of 34 PWH [22]. We observed an increase in CD4 cell counts in most patients, which may be attributed to both sustained viral suppression (reducing inflammation and CD4 damage associated with residual viremia) [23] and a potential intrinsic immunological benefit of CAB/RPV-LA, warranting further investigation [24]. Finally, we did not observe any significant or severe adverse events in our patients, apart from injection-site reactions. The SOLAR study, which compared CAB/RPV-LA with BIC/TAF/FTC, likewise reported no severe adverse events in the CAB/RPV-LA group (*n* = 454). The only drug-related adverse events, occurring in 2–3% of individuals, were pyrexia, headache, fatigue, and diarrhea [25].

## 5. Conclusions

In conclusion, CAB/RPV-LA represents a promising treatment option in challenging clinical settings, particularly for patients with poor adherence, gastrointestinal conditions affecting drug absorption, or persistent low-level viremia. Although our findings are based on a limited follow-up period and a small case series, they suggest that CAB/RPV-LA may be a feasible and effective strategy even in the absence of complete virological suppression. Further prospective studies are warranted to better define the role of CAB/RPV-LA in viremic individuals. The clinical experiences described herein may offer practical insights for clinicians facing similar therapeutic challenges in real-world settings.

## Figures and Tables

**Table 1:** Features of patients with unsuppressed HIV viral load switched to CAB/RPV-LA.

Patient	Site	Sex, Age (Years)	ART Before CAB/RPV-LA	Cause of the Switch	VL (Copies/mL) Before Switch	CD4 (cell/ mm^3^ and %) Pre and Post LA	Outcome	Follow-Up Duration (Months)
Case 1	Palermo	M, 54	DRV/c/TAF/FTC	IBD that compromised the absorption of his oral ART	84	From 557 (17%) to 759 (20%)	Sustained suppressed VL (<20 copies/mL)	8
Case 2	Palermo	M, 43	DRV/c/TAF/FTC	Poor adherence to oral therapies associated with psychosocial challenges, including the impact of stigma	2300	From 301 (19%) to 472 (25%)	Sustained suppressed VL (<20 copies/mL)	4
Case 3	Palermo	F, 63	DTG/ABC/3TC	Poor adherence associated with intolerance for oral therapies	18,900	From 330 (18%) to 337 (22%)	Sustained suppressed VL (<20 copies/mL)	12
Case 4	Palermo	M, 37	DTG + TDF/FTC	Low-level viremia	209	From 758 (41%) to 958 (43%)	Sustained suppressed VL (<20 copies/mL)	6
Case 5	Palermo	M, 38	BIC/TAF/FTC	Low-level viremia	84	From 373 (39%) to 460 (44%)	Sustained suppressed VL (<20 copies/mL)	3
Case 6	Palermo	M, 49	ATZ + TAF/FTC	Poor adherence to oral therapies due to side effects, including psychiatric symptoms	1940	From 565 (33%) to 511 (34%)	Sustained suppressed VL (<20 copies/mL)	7
Case 7	Rome	M, 59	DTG + DRV + r	“Pill fatigue”, poor adherence, and low-level viremia	86	From 166 (24%) to 405 (27%)	Sustained suppressed VL (<30 copies/mL)	31
Case 8	Rome	F, 52	RAL + TDF/FTC	Poor adherence and social instability	46	From 476 (25%) to 755 (22.6%)	Sustained suppressed VL (<30 copies/mL)	12

3TC: lamivudine, ABC: abacavir, ART: antiretroviral therapy, ATZ: atazanavir, BIC: bictegravir, c: cobicistat, CAB: cabotegravir, DRV: darunavir, DTG: dolutegravir, F: female, FTC: emtricitabine, IBD: intestinal bowel disease, LA: long acting, M: male, r: ritonavir, RAL: raltegravir, RPV: rilpivirine, TAF: tenofovir alafenamide, TDF: tenofovir disoproxil, VL: HIV viral load.

## Data Availability

Data are available on request to corresponding authors.

## Abbreviations

The following abbreviations are used in this manuscript:

3TC	Lamivudine
ABC	Abacavir
ART	Antiretroviral therapy
ATZ	Atazanavir
AZT	Zidovudine
BIC	Bictegrvair
c	Cobicistat
CAB	Cabotegravir
DOR	Doravirine
DRV	Darunavir
DTG	Dolutegravir
FTC	Emtricitabine
GRT	Genotyping resistance test
HIV	Human immunodeficiency virus
IBD	Inflammatory Bowel Disease
LA	Long-acting injectable
NGS	Next generation sequencing
RAL	Raltegravir
RPV	Rilpivirine
TAF	Tenofovir alafenamide
TDF	Tenofovir disoproxil
TDM	therapeutic drug monitoring
PWH	People with HIV
